# Supplementary material for: Are carbon emissions trading and green financial instruments synergistic? -Comprehensive quantitative research based on content analysis
Source: PLoS One. 2024 Mar 7;19(3):e0298601. doi: 10.1371/journal.pone.0298601 (PMC10919601; doi:10.1371/journal.pone.0298601)
Supplement: S1 Data — (ZIP) [file pone.0298601.s001.zip › Data/地级市Z_F工作报告.zip!/╔╜╢1⁄2/┴─│╟╩╨/└┤╫╘╣1⁄2╓┌║┼í╛┬φ┐╦╩2╛▌═°í┐_2013.docx]

各位代表：

　　现在，我代表市人民政府向大会报告政府工作，请予审议，并请市政协委员提出意见。

　　一、2012年工作的回顾

　　2012年是我市抢抓发展机遇、全力争先进位，在复杂形势下营造新优势、实现新突破的一年。一年来，在省委、省政府和市委的正确领导下，全市上下深入贯彻党的十八大、省十次党代会和市十二次党代会精神，紧紧围绕“全面建设生态型强市名城、创造聊城人民幸福生活”的奋斗目标，以开展“争先进位年”活动为动力，解放思想，开拓创新，真抓实干，圆满完成了市第十六届人大一次会议确定的目标任务。

　　（一）抓机遇，拓空间，聊城成为国家和省重大区域战略叠加区。我们着眼于培植聊城战略优势，抢抓机遇，积极争取，融入国家和省重大区域发展战略取得了历史性突破。我市成功加入中原经济区，被列为重要节点城市，一批重大基础设施和产业项目列入规划。融入省会城市群经济圈工作取得重大进展，省委、省政府对聊城的支持力度进一步加大。省十次党代会作出了“打造山东西部新的经济隆起带”的战略部署，前期调研论证工作已经完成，发展规划正在制定。各位代表，通过我们坚持不懈的努力，聊城已经成为国家和省重大区域发展战略叠加区，迎来了新的历史性机遇。

　　（二）抓投入，稳运行，全市经济保持又好又快发展。我们坚持把抓投入与稳增长相结合，预计完成规模以上固定资产投资1260亿元，增长21.8%。200个重点项目完成投资803亿元，比上年多完成288亿元，其中鲁西化工年产48万吨三聚氰胺、鑫亚电控燃油喷射系统等70多个项目建成投产、发挥效益。基础设施建设取得新的突破，火车站改造及站前广场、高临高速已经竣工，邯济铁路扩能改造、德商高速聊城段、国家公路运输枢纽、济聊一级公路加快建设，青兰高速聊城段前期工作进展顺利，郑济快速铁路、聊泰铁路、军民合用机场争取工作取得新的进展。在抓投入的同时，精心组织经济运行，帮助企业解决困难，经济保持了又好又快发展。预计全市生产总值完成2150亿元，增长12.5%；地方财政收入104.5亿元，按可比口径增长18%；规模以上工业主营业务收入6500亿元，利税650亿元，分别增长25%和26%；社会消费品零售总额722亿元，增长14.6%；城镇居民人均可支配收入23800元，农民人均纯收入9088元，分别增长15.3%和17.5%，全市绝大多数指标增幅继续位居全省前列。

　　（三）抓调整，促转变，各产业发展格局进一步拉开。我们坚持扩总量与转方式、调结构相结合，以建设“一五二”产业基地为重点，努力拉开发展格局、培植发展优势。一是农业产业调整振兴成效显著。预计粮食总产116亿斤，实现“十连增”；瓜菜菌总产1440万吨，保持全省第一；畜牧和水产、林果生产及加工产值分别达到144亿元和186亿元。新增省级龙头企业9家、农民专业合作社739个、“三品”认证品种75个。聊城农产品物流交易中心一期基本完成。南水北调聊城段主体基本完工，灌区续建配套与节水改造、国家小农水重点县项目进展顺利，中小河流治理全面推进，新建、修复机井9222眼，完成农田水利建设投资10.6亿元，增长47%。农机总动力达到1150万千瓦，增长6%。二是工业“4455”工程加快实施。四大战略性支柱产业、四大战略性新兴产业、五大民营特色产业和50户骨干龙头企业主营业务收入分别增长18%、20%、27%和23%。“3+4”千亿产业园建设全面展开，累计完成公共设施投资150亿元，工业投资840亿元，建成区面积达到55平方公里，阳谷祥光生态工业园顺利通过国家级生态工业示范园区验收。三是服务业发展步伐加快。狠抓服务业重点项目建设和支持政策落实，举办了首届“水文化”节和“好客山东”休闲汇等活动，我市被评为“中国温泉之城”。预计服务业投资完成350亿元，用电量增幅连续两年高出全省平均水平10个百分点，增加值占生产总值比重达到31%。新引进商业银行2家、村镇银行4家，金融机构本外币存贷款余额分别达到1687.7亿元和1289.2亿元。国内旅游收入增长28%。四是科技创新和节能减排扎实推进。新增省级企业技术中心9家，牵头组建省级产业技术创新战略联盟2家，高唐被列为国家火炬计划特色产业基地，“智慧聊城”建设全面启动，预计高新技术产业产值占规模以上工业比重提高2个百分点。新增中国驰名商标1个、山东省名牌10个，时风集团获得省长质量奖。中冶纸业启动了全省首个中德能效合作项目，淘汰关闭落后产能企业13家，实施大气和水污染减排工程57项，节能减排目标可全面完成。各位代表，我们在强化农业基础的同时没有影响工业发展，工业化在快速推进的同时实现了节能减排工作提升，在经济欠发达的情况下做到了民生持续大幅改善。去年10月份，中国生态文明建设国际论坛在我市成功召开，我市经济发展与环境保护互促共赢的发展模式被誉为生态文明“聊城现象”，这是国内外领导和专家对我市的肯定，是全市人民的光荣！

　　（四）抓城建，促统筹，城乡面貌发生新的变化。我们坚持把城镇化作为支撑工业化、改善群众生活的重要途径，进一步加大了城镇规划建设管理力度。江北水城建设开创了新的局面， 20个城建重点项目完成投资102亿元，增长45.7%。古城保护与改造四条大街临街建筑和道路、综合管沟等基础设施基本完工；徒骇河世界运河（建筑）博览园滨河大道大部分通车，防洪治理二期主体工程基本完工；市民文化活动中心、西安交大聊城科技园学术交流中心主体完工；体育公园全民健身中心投入使用，体育场主体基本完成；北部物流园区、开发区中央商务区等项目建设也都取得很大进展。以道路为重点的市政基础设施建设取得新的突破，54条主次干道和背街小巷拓宽改造全面完成，进一步方便了群众出行；城区新增供热面积117万平方米、管道天然气1.47万户，开通校车专线20条，居民生活条件进一步改善。城市管理进一步加强，环境综合整治行动、“退路进市”工程取得明显成效。各县（市）城区和小城镇建设也都取得很大进展，茌平、东阿、高唐被评为省级园林城市，14个示范镇建设全面启动。坚持城乡统筹，全面推进新农村建设，路域环境综合整治取得阶段性成果，新改建农村公路1790公里，市到县、县到乡的客运线路公交化改造全面完成，31万农村群众饮水安全问题得到解决；建设农村新居4.6万户，改造农村危房2.1万户，启动千户社区126个，农村居民生活环境进一步改善。

　　（五）抓改革，促开放，经济社会发展活力明显增强。我们坚持把改革开放作为强大动力，坚定不移地加以推进。国有企业改革继续深入，经营预算管理进一步完善，活力明显增强。新增股权挂牌企业10家，直接融资13.6亿元。农村土地流转新机制基本形成，新增流转面积7.86万亩；集体林权制度配套改革深入推进。大力推进民营经济争先进位，民营经济税收、民营工业固定资产投资分别增长20.7%和20%。国库集中支付改革进一步深化，公务卡改革全面启动，专项资金管理进一步规范。事业单位分类改革稳步推进。组织了春、秋两季招商引资“百日会战”，招商引资到位资金500亿元，增长23.5%；山东省和韩国京畿道第一个省道合作产业园区落户市经济开发区，阳谷电缆与法国耐克森公司成功合作，全市实际利用外资1.1亿美元，完成进出口总值56亿美元。“海外聊城”建设取得新进展，新批境外企业7家，境外投资4500万美元，获得国外铝土资源开采权8960万吨、铜勘探开发权面积1万平方公里。

　　（六）抓民生，促和谐，人民生活有了新的改善。我们始终坚持把保障改善民生作为工作的出发点和落脚点，全市财政用于民生的支出达到147.6亿元，占财政总支出的68%，10件民生实事全部落实。预计城镇新增就业7万人，其中失业人员再就业2.9万人，困难群体就业5500人，城镇登记失业率为3.1%。社会保障体系进一步完善，“五七工”、“家属工”、未参保集体企业退休人员养老保障问题基本解决，企业退休人员养老金、城乡低保标准、城镇居民医疗和新农合政府补助标准全面提高。保障性安居工程建设进展顺利，新开工各类保障性住房1.5万套，竣工8163套，开工率、竣工率均居全省前列，政府限价房完成配售1208户。教育条件明显改善，教学质量进一步提高，开工建设乡镇中心幼儿园61所；新建、改建农村中小学校舍31.2万平方米，农村中小学贫困寄宿生补助标准提高了500元，336所中小学热水热饭、144所学校取暖问题得到解决；城区新建小学3所,新增教室138个，新招班额控制在50人以下;聊城职业技术学院成为省首批技能型人才培养特色名校，高级工程职业学校、高级财经职业学校新校区26栋单体建筑主体竣工。文化事业加快发展，国有文化站馆向社会免费开放，聊城市豫剧院剧场建成使用。成功举办了全市第二届全民健身运动会，群众性体育活动更加深入普及。卫生改革发展取得新的成效，公立医院改革稳步推进，东阿县走在全省前列；纳入省统一规划的村卫生室全面实施了基本药物制度，药物价格平均下降36%；“农民健康服务行动”为边远地区群众诊治疾病、健康体检15.5万人次，广大农民为此节省医疗费用520多万元。社会救助工作进一步加强，高标准的市儿童福利院正式启用，医疗救助“一站式”结算平台全面开通，“明天计划”、“重生行动”、先心病手术项目进展顺利。“百村扶贫攻坚”取得明显进展，预计贫困村农民人均纯收入增长19.5%，高于全市平均水平2个百分点。食品药品安全形势总体稳定，安全生产事故起数、伤亡人数和直接经济损失全面下降，十八大安保任务全面完成，打霸治痞取得显著成效，社会保持和谐稳定。国防动员、民兵预备役、人民防空、双拥共建和优抚安置工作取得明显成效，新闻出版、社会科学、人口计生、妇女儿童、青少年、残疾人、慈善、老龄等事业持续发展，统计、档案、史志、气象、油区、对台、防震减灾、外事侨务、民族宗教等工作都取得了新的成绩。

　　（七）抓作风，促落实，政府自身建设进一步加强。我们坚持以转变作风、狠抓落实为重点，深入开展“争先进位年”活动和“塑造聊城形象从我做起”主题活动，全市上下展现出全新的精神风貌。认真落实政府工作推进“八项制度”，在重点项目建设、园区和开发区建设、民营经济发展、县域经济发展等方面出台了考核奖惩办法，对137个单位开展了重大事项落实情况督查，对67个单位及所属系统进行了政风行风检查，狠抓落实之风进一步形成。全面推进依法行政，办理人大代表建议和政协委员提案281件。扎实开展行政程序年活动，修订了《聊城市人民政府工作规则》，实行了政府法律顾问制度、规范性文件“三统一”制度，开展了行政复议委员会试点，政府工作的规范化水平明显提升。进一步转变政府职能，取消、下放市级行政许可40项，取消、暂停行政事业收费39项，市行政服务中心集中办理事项提高到90.3%。深入推进政务公开，我市被评为中国城市信息化50强，市长公开电话承办事项群众满意率达到87.5%。应急管理体系进一步完善，应对突发事件能力明显增强。强化行政监察和审计监管，严肃查处了一批违法违纪案件。

　　各位代表，过去的一年，我们面临的形势复杂严峻，世界经济复苏明显放缓，国内经济下行压力加大，我市改革发展稳定的任务十分艰巨。在这种情况下，我们正确处理各方面关系，实现了经济又好又快发展、各项民生显著改善、社会保持和谐稳定，是十分不容易的。这是中央、省委省政府和市委正确领导的结果，是人大、政协和社会各界监督支持的结果，更是全市上下同心同德、奋力拼搏的结果。在此，我代表市人民政府，向全市工人、农民、知识分子，向所有企业家和纳税人，向各民主党派、工商联、各人民团体，向驻聊人民解放军、武警官兵、公安干警和中央、省驻聊单位，向所有关心、支持聊城发展的海内外朋友，表示衷心感谢！

　　各位代表，在看到成绩的同时，我们也清醒地认识到，虽然近年来全市经济社会快速发展，但经济总量小、财政实力弱、城镇化水平低的问题依然存在，加快发展的任务仍然十分艰巨。特别是与十八大精神的要求相比，我市经济发展的质量、改革开放的力度、创新驱动的能力、社会建设的水平还存在很大差距，转方式、调结构、惠民生的压力巨大。对此，我们一定要认真进行研究，扎实做好工作，努力推动聊城经济社会又好又快发展。

　　二、2013年政府工作的任务目标

　　各位代表，今年是全面深入贯彻十八大精神的开局之年，是实施“十二五”规划承前启后的关键一年，是为全面建成小康社会奠定坚实基础的重要一年。做好今年的政府工作，意义十分重大。

　　今年，我们面临的国内外环境依然复杂，世界经济低速增长态势仍将延续；我国经济趋于稳定，积极因素进一步增多，但面临的困难仍然较多。对此，我们必须保持清醒头脑，坚持“底线思维”，从最坏处着眼，向最好处努力。同时，我们更要看到，我市经济社会发展面临着新的发展机遇和有利条件。一是我国发展仍处于重要战略机遇期，中央继续实施积极的财政政策和稳健的货币政策，国内经济发展的基本面将持续向好。二是我市加入中原经济区，省委、省政府加快建设山东西部新的经济隆起带和省会城市群经济圈，我市将获得国家和省越来越多的重视和支持。三是通过近年来我们坚持不懈地抓招商、抓项目、抓调整，我市产业发展的格局全面拉开，城市建设打开新的局面，基础设施更趋完善，加快发展的基础更加稳固。四是通过党委、政府换届，开展“争先进位年”活动，特别是学习贯彻十八大精神，全市上下科学发展、加快发展的热情和干劲空前高涨！我们坚信，只要全市上下同心同德、抢抓机遇、扎实苦干，我们就一定能够战胜任何艰难险阻，在建设生态型强市名城的征程上迈出新的更大的步伐！

　　2013年政府工作的总体要求是：深入学习和全面贯彻党的十八大精神，坚持以邓小平理论、“三个代表”重要思想、科学发展观为指导，按照市第十二次党代会的部署，紧紧围绕主题主线，牢牢把握稳中求进的工作总基调，以建设“一五二”产业基地为总抓手，坚持“四市方针”和“三个城市定位”，着力加大优质高效投入，着力深化改革开放，着力强化创新驱动，着力保障改善民生，全面协调推进新型工业化、信息化、城镇化、农业现代化步伐，努力实现经济又好又快发展和社会和谐稳定。主要预期目标是：全市生产总值增长11%，地方财政收入增长15%，规模以上固定资产投资增长21%，进出口总额增长10%，实际利用外资增长15%，社会消费品零售总额增长15%，城镇居民人均可支配收入、农民人均纯收入分别增长12%和14%，城镇登记失业率控制在3.8%以内，居民消费价格涨幅控制在3.5%左右，人口自然增长率控制在7‰以内，节能减排等约束性指标完成省下达的任务。

　　各位代表，全面实现2013的任务目标，必须统筹安排、科学把握。一是坚持速度、质量、效益相统一。坚持扩总量与“转、调、创”相结合，既要保持较快的增长速度，确保主要指标增幅高于全省平均水平、高于周边市；又要着力提高经济增长的质量和效益，做到好字当头、好中求快、又好又快。二是坚持改革、发展、稳定相协调。既要着眼聊城科学发展、跨越发展，进一步深化各项改革，千方百计加快发展步伐，又要把改革的力度、发展的速度与社会的可承受程度结合起来，实现改革发展稳定相协调。三是坚持工业化、信息化、城镇化、农业现代化统筹推进。坚持以工业化为主导，推动工业化与信息化深度融合、工业化与城镇化良性互动、城镇化与农业现代化相互协调，加快形成以工促农、以城带乡、工农互惠、城乡一体的发展格局。四是坚持增投资、扩消费、惠民生同步发展。坚持把加大优质高效投入作为实现聊城科学发展、跨越发展的关键措施，紧紧围绕扩消费、惠民生优化投资结构，寻求投资与消费的结合点，使发展成果更多、更公平地惠及全市人民。

　　三、加快构建现代产业体系

　　构建现代产业体系，是转方式、调结构、保增长的必然要求，更是强实力、增效益、惠民生的根本要求。要立足当前、着眼长远，用全局的眼光、战略的思维，加快构建既能增强竞争力、又能支撑聊城未来发展的现代产业体系。

　　（一）加快构建现代农业产业体系。围绕打造生态农业及农产品深加工基地，以聊城农产品物流交易中心为龙头，加快规划建设与之相配套的农产品生产基地体系、农产品加工龙头企业体系、专业市场及信息服务体系、农民专业合作社体系、农产品安全保障体系、农机水利科技服务等体系，力争用3-5年时间，构建起符合国情市情的现代农业大的框架体系，提高农民收入水平，为广大农村全面建成小康社会奠定坚实基础。今年重点抓好四个方面：一是稳步提高农业综合生产能力。继续抓好全国新增千亿斤粮食产能规划和山东省千亿斤粮食产能规划聊城项目区建设，大力推进整建制高产创建，全面落实种粮直补、良种补贴、生产资料补贴政策，确保粮食总产稳定在110亿斤以上，力争实现“吨粮市”目标。大力发展设施农业，力争蔬菜总产达到1500万吨以上。二是实施农产品品牌战略。围绕蔬菜和食用菌、畜牧和水产、林果生产及加工三大支柱产业，抓好100个标准化示范基地、67个畜禽养殖标准化示范场建设。利用物联网信息化通道，加快构建农产品从生产到市场的全程质量控制系统和追溯制度，力争培育知名农产品区域公用品牌10个、国家地理标志产品和注册商标10个，新增“三品”认证品种60个以上。三是大力推进农业产业化。加大对农业龙头企业的扶持力度，继续抓好100家农民专业合作示范社，着力提高经营水平。发挥供销社和邮政服务“三农”的作用，鼓励工商企业投资农业，积极推进农超对接、农企对接、农餐对接。四是加强农业基础设施建设。抓好南水北调聊城段建设，确保如期通水，加快推进8座配套平原水库建设。推进灌区续建配套与节水改造，新增节水灌溉面积18万亩。全面落实农机具购置补贴政策，主要农作物机械化水平达到85%以上。

　　（二）加快构建现代工业产业体系。围绕建设五大工业产业基地，以打造山东西部新兴生态化工业城市为目标，以“4455”工程为抓手，着力扩张总量、优化结构、塑造特色，力争到“十二五”末，全市规模以上工业主营业务收入突破1万亿元大关。一是大力培植战略性支柱产业。继续下大力抓好铜及铜加工、铝及铝加工、新能源汽车和精细化工等四大战略性支柱产业发展，使其尽快成为支撑聊城现代工业发展的战略支柱。把“千亿产业园区”作为培植战略性支柱产业的主要载体，以创建国家级示范园区为方向，集中力量抓好“3+4”千亿产业园区建设，确保“十二五”期间祥光生态工业园、信发循环经济产业园和鲁西化工新材料产业园主营业务收入均达到1000亿元以上；2020年前，新能源汽车、泉林循环经济、临清轴承、莘县油盐化工等4个园区主营业务收入达到1000亿元以上。二是加快培育战略性新兴产业。加大对新能源、生物医药、节能环保和高端装备制造等产业的支持力度，加快西安交大聊城科技园、九州国际高科园和高新技术创业服务中心建设，高新技术产业在规模以上工业中的比重再提高2个百分点。三是加大开发区建设力度。把开发区作为构建现代工业产业体系的战略支撑，努力拉开框架、培育特色，争先进位、跨越发展，9个省级开发区地方财政收入、固定资产投资增幅不低于30%。四是突出抓好民营工业、龙头企业和市属企业发展。以轴承、冶金板材、钢管、钎具、畜禽加工等五大民营特色产业为重点，规划建设产业园区，积极搭建服务平台，在扩张规模的同时进一步提升发展水平。加大对50户骨干龙头企业的扶持力度，鼓励企业引进配套企业、领办产业园区、延伸产业链条，变企业优势为产业优势。实施市属企业“翻番计划”，推动市属工业企业在“十二五”期间实现主要指标翻番，今年主营业务收入突破500亿元，地方财政贡献达到7亿元。五是推进信息化和工业化深度融合。把信息化作为构建现代工业产业体系的重要支撑，深入实施“十百千”工程，抓好信息化示范企业、实验园区和两化融合共用平台建设，引导制造业向智能制造和服务型制造转型升级，加快建设“智慧聊城”。

　　（三）加快构建现代服务业体系。加快服务业发展是我市转方式、调结构、增效益的关键。围绕建设商贸物流及现代物流、文化旅游及休闲度假两大基地，突出抓好100个服务业重点项目建设，力争服务业在三次产业中的比重提高2个百分点。一是大力发展商贸物流业。进一步加快北部物流园区、农产品物流交易中心建设，尽快形成商贸物流中心城市的支撑扩张点。大力发展新型物流业态，努力扩大商贸物流规模。二是着力突破文化旅游业。以打造江北文化旅游和休闲度假目的地城市为目标，以历史文化、温泉休闲、阿胶养生为特色，以“一湖三河”为重点，突出抓好中华水上古城、阿胶养生文化苑等20个重点项目建设。实施湖河灯光工程，打造“夜游水城”精品线路。继续举办“水文化”节系列活动，打造大型演艺项目，进一步提升聊城的知名度和文化旅游吸引力。三是全面抓好其他服务业发展。积极引进辖外金融机构，加强地方金融改革创新，优化金融生态环境，建立健全科学防范化解金融风险的管理体制，全面促进金融产业做大做强。充分发挥我市医疗、教育等资源优势，积极发展新型家政服务。努力促进房地产、农村服务、社区服务、中介服务等服务业健康快速发展。

　　（四）坚持不懈地加大优质高效投入。构建现代产业体系，加大投资力度是根本，优化投资结构是关键。一是认真抓好200个市级重点项目建设。力争完成投资1000亿元，重点抓好鲁西化工新材料、泉林150万吨秸秆造纸、信发集团离子膜烧碱等大项目好项目建设。加速推进交通基础设施项目建设，邯济铁路聊城段扩能改造全面完工，德商高速聊城段、国家公路运输枢纽、济聊一级公路建设全面提速，青兰高速聊城段、郑济快速铁路、聊泰铁路、军民合用机场加快前期工作，争取早日开工建设。二是下决心调整优化投资结构。在确保战略性支柱产业投资的同时，采取有效措施引导更多的资金投向战略性新兴产业、现代服务业、现代农业和民生事业。调整财政投入结构，重点支持引领区域发展重大项目、县乡财源支撑项目和优质高效高端产业项目。严格执行国家产业政策，严格限制投资强度低、财政贡献少、能耗排放高的项目。三是努力破解发展瓶颈。加强银企合作，积极发展企业上市、股权托管交易、企业债券发行、融资租赁等直接融资方式，增加并引导好民间投资，力争新增上市公司1家、股权挂牌企业10家，社会融资规模达到360亿元。抓好土地增减挂钩政策落实，依法开展土地挖潜改造，盘活企业用地2万亩，努力为项目建设创造良好条件。

　　（五）大力实施创新驱动发展战略。坚持把创新驱动摆在现代产业体系建设的核心位置，加快构建以企业为主体、市场为导向、产学研相结合的技术创新体系，全面推进科技创新、产品创新、品牌创新、产业组织创新和商业模式创新，不断增强经济发展内在活力和市场竞争力。支持企业创新平台建设，鼓励企业牵头实施重大科技项目，新增省级以上工程技术研究中心5家、企业技术中心4家，实施企业技术创新项目70项。继续组织开展科技合作洽谈活动，推动企业加强与高校院所的合作。抓好科技成果推广中心、技术市场建设，推进科技成果推广应用。实施质量强市和品牌战略，抓好鲁西质检中心和120个技改项目建设。大力推进科技兴农，重点抓好10个农业科技创新平台、20个农业科技示范园区建设，健全完善基层农技推广、动植物疫病防控、农产品质量监管等服务体系。大力实施人才强市战略，抓好博士后科研工作站、引智示范基地建设和“533”高端人才引进，加强星火科技培训，支持科技人才以科研成果、专利权等无形资产入股，努力把科技人员科技报国的积极性充分发挥出来。

　　四、加快推进新型城镇化

随着全市经济的发展，特别是工业化的推进，加快新型城镇化已经成为全市经济社会发展和全面建成小康社会的战略任务。要从实际出发，统筹规划，分步实施，努力加快新型城镇化进程，力争城镇化率每年提高2个百分点。

　　（一）中心城市率先发展。中心城市是全市城镇化的龙头。加快新型城镇化进程，中心城市必须率先发展。要以建设“双百”大城市为目标，进一步拉大城市框架，突出水城特色，完善城市功能，增强城市竞争力。一是着眼于提升聊城在省会城市群经济圈中的地位，提高规划水平。充分发挥规划的引领作用，加快修订总体规划，完善城区水系、道路交通等专项规划，超前做好徒骇河、马颊河、南部新区等控制性详规和重点项目规划储备，建成区详规覆盖率达到100%。二是按照“一年一大变，三年大变样”的要求，抓好重点城建项目建设。徒骇河滨河大道、市民文化中心、体育公园体育场、西安交大聊城科技园等全面完成；古城保护与改造工程全面完成沿街建筑，恢复部分历史景观，力争年内实现运营；徒骇河世界运河（建筑）博览园实施景观绿化和防洪治理。对重点城建项目，建立工作推进、环境保障、督查落实“三位一体”工作机制，形成整体合力，确保工作成效。三是着力塑造水城特色，提高城市宜居水平。实施湖河水系工程，启动运河五期和南湖水库建设，开展东南湖沿岸整治。实施城市“绿荫行动”，抓好节点公园、主次干道绿化工作，提升园林绿化水平。加快生活垃圾焚烧发电厂、中水回用工程建设，确保竣工运行。四是坚持城市管理与建设并重，提高城市管理水平。实施城市综合整治，开展“和谐城管”创建活动，加强便民市场建设管理，坚决查处违规建设。各位代表，在城市管理上，我们坚持以人为本，对群众的合理诉求，我们要最大限度地予以满足；对少数的不合理诉求，我们要认真向群众解释说明；但对于乱搭乱建、套取国家资金的行为，我们必须坚决取缔、毫不含糊！

　　（二）各县（市）城区加快推进。各县（市）城区是全市城镇化的重要支撑，对县域城镇化具有重要的辐射带动作用，必须加快推进。各县（市）要突出抓好经济开发区、工业园区和交通干线沿线城郊隆起带建设，加快棚户区改造，进一步膨胀城市规模。高水平编制城市规划，发挥各自优势，突出城市特色。结合平原水库建设和湖河治理，突出水系、生态，加快构建“大水城”格局。东阿、茌平要积极与中心城市对接，共同构建“大三角”地带，主动承接济南辐射，增强聊城在省会城市群经济圈中的影响力和竞争力。积极推进户籍制度改革，破除农村人口向城镇合理流动的壁垒。

　　（三）小城镇建设重点突破。加快新型城镇化进程，小城镇是关键。把小城镇作为加快新型城镇化的重要支撑，以示范镇建设为重点，加快推进，全面突破。抓好小城镇规划编制工作，着力提升规划水平，基本实现全覆盖。实施重点带动，适时出台支持政策，加快5个省级、9个市级示范镇的发展。鼓励示范镇解放思想、创新思路，积极探索市场化开发建设的路子，敢于和善于破除制约小城镇发展的体制机制障碍，增强自我发展能力。把示范镇建设与产业发展、园区建设统筹考虑、统一规划、一体推进，增强产业支撑。同时，鼓励其他乡镇创造性地开展工作，加快建设和发展步伐。

　　（四）农村新居建设和危房改造常态化。改造危房、建设新居是广大农民的普遍愿望，也是推进新型城镇化的重要内容，要在前三年工作的基础上，继续抓紧抓好。加强对农村新居建设的规划、指导和服务，避免出现10年、20年重建一次的现象。要以建设农村新型社区为重点，在尊重农民意愿的前提下，引导农民向交通、教育、卫生、供水、供电等配套设施相对齐全的社区集中，规划整合农村社区150个。推广“强村兼并”、“企业带动”等模式，鼓励有条件的企业建设农村社区，推进工农城乡一体化发展。各位代表，我们要尽最大的努力，使越来越多的父老乡亲从低矮破旧的房屋搬到宽敞明亮的新居，享受类似城市居民的生活！

　　五、着力深化改革扩大开放

　　各位代表，我国最近30多年取得的巨大成就靠的是改革开放，要实现聊城科学发展、跨越发展，更需要大力推进改革开放。

　　（一）深入推进体制改革。加快推进国有企业和国有资产管理体制改革，鼓励企业对外开展战略合作、对内进行整合重组。深化农村土地管理制度和产权制度改革，完成农村集体土地所有权、农村宅基地使用权和其他建设用地使用权确权登记发证工作。积极培育农村产权流转市场，规范推进土地承包经营权流转。抓好财税改革，完善税收征管和财政资金监管制度，强化部门预算约束，提高资金使用效益。积极稳妥地推进事业单位改革，做好事业单位分类工作。

　　（二）大力发展民营经济。民营经济在我市科学发展跨越发展、缩小与发达市差距中不可或缺，事关全市经济社会发展大局。要实施“11236”工程，强力推进民营经济加快发展，力争“十二五”末达到全省中等以上水平，今年民营经济投资达到680亿元，税收增长20%以上。着力培育创业主体，鼓励全民创业，新增个体工商户和私营企业2万户左右。加大对民营经济龙头企业的支持力度，对获得国家和省名牌产品称号的企业予以奖励。抓好窗口服务平台建设，搞好中小企业融资、科技、信息服务，整治涉企乱收费、乱摊派、乱罚款等不良行为，努力为民营经济发展营造良好的环境。

　　（三）全面抓好对内对外开放。进一步加大招商引资、招才引智力度，突出招大引强，突出项目质量，突出项目落地，重点抓好优势产业招商、特色园区招商、重点企业招商和以商招商，确保招商引资到位资金达到600亿元。实施技术、品牌、营销、服务“四带动”出口战略，抓好6个省级外贸出口基地和出口农产品质量安全示范区建设。积极增加进口，鼓励企业引进先进技术、装备和关键零部件，促进产业转型升级。进一步提高口岸通关效率，积极帮助企业防范和抵御国际经济风险。大力实施“海外聊城”战略，以非洲、南美为重点，鼓励企业走出去开展资源开发和加工贸易；加强外派劳务资源整合，抓好3个对外劳务平台建设，争取境外工程承包实现突破。

六、深入推进生态文明建设

　　各位代表，我市是一个资源消耗大市，也是一个正在崛起的新兴工业城市，加强生态文明建设是我们长抓不懈的一项重要任务。我们要坚持把生态文明理念贯穿于经济社会发展全过程，巩固和发展好生态文明“聊城现象”，努力建设美丽聊城。

　　（一）全面推进资源节约。这是我市转方式、调结构重大而紧迫的任务。突出抓好重点领域、重点产业、重点企业节能工作，组织实施20项节能重点工程，支持节能低碳产业和新能源、可再生能源发展。实行最严格的耕地保护和节约用地制度，依法加强土地执法管理。推广“1062”循环经济模式，抓好循环经济示范园区建设，推进减量化、再利用。

　　（二）切实抓好环境保护。坚持预防为主、综合治理，全面推进工程减排、结构减排和管理减排，确保主要污染物持续均衡削减。以大气和水污染治理为重点，加快燃煤电厂脱硝工程建设和脱硫设施运营监管，开展化工、食品、制药等行业和污水处理厂异味治理。加快构建以人工湿地建设为核心的水污染防治体系，彻底解决污水直排环境问题，推进河流水质持续改善。狠抓环境质量监测管理，加快构建环境预警监测体系。做好代表山东省迎接国家海河流域水污染防治核查工作，确保再次取得优异成绩。

　　（三）持续加强生态建设。围绕生态宜居、美丽聊城，抓好“绿色学校”、“绿色社区”、“绿色家庭”创建活动，营造“保护环境、人人有责”浓厚氛围。大力开展植树造林，抓好高速公路沿线绿色通道和南水北调沿线生态建设，完成造林面积10.7万亩，新建和完善农田林网100万亩，力争森林覆盖率达到32%。实施农村环境连片整治，开展集中式饮用水源地保护和农村生活污水集中处理试点，积极推进农村垃圾村收集、乡运输、县处理，抓好秸秆综合利用和沼气、太阳能等新型能源推广，进一步改善农村生态环境。

七、切实抓好民生和社会建设

各位代表，政府是人民的政府，人民对美好生活的向往就是我们的奋斗目标。我们要始终把保障改善民生作为工作的出发点和落脚点，心中始终想着群众，尽心竭力为群众办实事、办好事，努力让全市人民过上更好生活。

　　（一）切实办好民生实事。我们要进一步加大民生投入，着力解决好人民群众最关心最直接最现实的利益问题。重点抓好“八大民生实事”：一是“人尽其力”取得新进展。坚持扩大就业与推动创业并重，加大政策扶持和培训力度，做好以高校毕业生为重点的青年就业工作，新增城镇就业6万人，其中帮扶困难群体就业4000人，新增农村劳动力转移就业5万人。适时提高企业工资指导基准线和最低工资标准，构建和谐劳资关系。二是“老有所养”实现新提升。统筹推进城乡社保体系建设，突出抓好农村转移就业人员、失业职工、无固定职业者等重点群体参保工作，继续增加企业退休人员基本养老金。抓好农村“五保”规范化管理，基本完成新老农保制度衔接。做好“幸福敬老院”创建工作，鼓励发展社会养老服务，开展居家养老、社区养老和机构养老，新增床位2390张。三是“学有所教”提升新水平。加快实施“学前教育三年行动计划”，建设乡镇幼儿园59所，所有乡镇中心幼儿园达到省定标准，建设市实验幼儿园。完成中小学校舍安全工程,“211”工程实现农村中心中学和寄宿制学校全覆盖；城区新建小学3所，新增教室120个，新招班额控制在45人左右；加强教师队伍建设，进一步提高教育质量。支持聊城大学、聊城职业技术学院分院分别建设省应用型、技能型人才培养特色名校，加强与山东工程技师学院的合作，支持东昌学院加快发展，确保聊城高级工程职业学校和高级财经职业学校新校区建成使用，促进民办教育健康发展。四是“病有所医”增加新保障。加快推进公立医院改革，全面提升医疗卫生服务能力和水平，打造省级区域医疗卫生服务中心。加强基层医疗卫生机构建设管理，强化医护人员教育培训，基本实现村卫生室和非政府办基层医疗机构基本药物制度全覆盖。扩大新农合重大疾病保障试点范围，大病医疗费用报销后，个人负担超过上年省农民人均纯收入部分，补偿比例不低于50%。进一步扩大“全民健康工程”覆盖面，继续实施农村贫困家庭儿童先心病免费治疗。扎实做好疾病防控工作，确保不出现传染病暴发流行。五是“住有所居”实现新扩展。坚持市场配置与政府保障相结合，开工建设保障性住房20835套、基本建成9000套，努力满足城镇中低收入家庭、新就业职工和进城务工人员基本住房需求。中心城市集中供暖率达到70%，两年内基本解决供暖问题；加大棚户区改造力度，全面完成规划设计和9个村改造任务。六是“出行通畅”迈出新步伐。中心城市新建改建主次干道24条，改造提升背街小巷49条，更新公交车50部，增加公交校车20部。完成临博路、临莘路改建工程和农村公路网化示范工程，改造农村公路1000公里，两年内基本解决危桥问题，推进村村通向村内通、网络化发展。七是“饮食安全”采取新举措。完善食品药品安全监管体制机制，扎实开展专项整治，实行举报奖励制度，全面提升安全水平。实施农村饮水安全工程，解决15万人饮水安全问题。八是“困有所帮”取得新成效。继续提高城乡低保标准，加大社会救助工作力度。深入实施“百村扶贫攻坚”计划，推进集中连片开发式扶贫，确保扶贫村农民人均纯收入增幅高于全市平均水平。各位代表，做好民生工作，经济是基础，目前我市经济发展水平还不高，但请大家相信，各级政府为人民群众办实事的态度是真诚的，我们一定尽最大努力把各项民生实事办好！

　　（二）大力推进文化强市建设。充分发挥聊城历史文化优势，突出重点，强化支持，改革创新，用三到五年的时间，建成符合中国特色社会主义文化发展方向、具有浓郁地方特色、具有较强影响力和竞争力的文化强市，真正将丰富的文化资源转变为现实生产力，将潜在的文化影响力转变为现实的城市竞争力。今年重点实施“五大工程”：一是特色文化打造工程。突出运河文化、水浒文化、阿胶养生、温泉休闲、书画艺术五大主题，规划建设一批文化产业项目、园区和示范基地，突出抓好运河文化大码头、北（京）聊（城）弘文书苑的策划和运作，加快形成聊城文化强市的亮点。以备战“十艺节”为契机，打造一批舞台艺术精品、群众文艺精品和美术精品。二是水城文化人才培育工程。组织文化人才在北京等大城市举办“江北水城书画精品展览”等活动，提升聊城文化的知名度，促进人才培养和相关产业发展。三是文化惠民普及工程。加快市民文化中心、市图书馆和县乡村公共文化设施建设，提升硬件设施和服务水平。开展“欢乐下基层”公益演出，积极培育农村文化，组织艺术院团进社区、进农村、进基层，全年演出400场以上。提升农村公益电影放映质量，分步实施农家书屋图书更新，推动公共文化设施免费开放。四是历史文化弘扬工程。加强对聊城历史文化典籍的收集、挖掘和整理，开展可移动文物普查，抓好非物质文化遗产保护传承。五是美丽聊城展示工程。围绕“美丽中国、美丽聊城”主题，采取“写、画、说、唱、摄”等形式，举办系列活动，使聊城的知名度、影响力、吸引力提升到新水平，进一步弘扬“自豪、创业、包容、奋进”的聊城精神！

　　（三）全面加强社会建设。社会建设是社会和谐稳定的重要保证。我们必须从维护广大人民群众根本利益的高度，加强和创新社会管理。进一步加强基层社会管理和服务体系建设，增强城乡社区服务功能。抓好人口和计划生育工作，开展基层基础年活动，加强流动人口管理服务和出生人口性别比治理，实施免费孕前优生健康检查，稳定低生育水平。加快公共体育设施建设，举办第二届全市运动会，提高群众健康水平。积极稳妥地推进殡葬改革，实现尊祖奉宗与合理利用土地的有机统一。完善落实优抚安置和退役士兵自主就业政策，增强全民国防观念，提高国防动员和后备力量建设质量。加强“一案三制”建设，进一步提高突发事件应急能力。坚持把安全生产作为重大政治责任，健全完善责任体系，强化监管措施，确保不发生重大安全事故。持续推进平安聊城建设，注重从源头上预防化解矛盾，加强社会治安综合治理，深入开展严打整治，保持社会和谐稳定。继续做好减灾救灾、社会科学、民族宗教、外事侨务、残疾人、慈善、对台、油区、人防、档案、史志、气象、测绘、防震等各项工作。

　　八、进一步加强政府自身建设

　　各位代表，面对全面建成小康社会的新目标和全市人民的新期盼，各级政府必须加快职能转变，推进管理创新，努力建设职能科学、结构优化、廉洁高效、人民满意的服务型政府。

　　（一）切实改进工作作风。认真贯彻落实中央、省委和市委关于改进工作作风、密切联系群众的规定和要求，从群众反映最强烈的问题入手，认真进行整改。坚持轻车简从，经常深入基层开展调查研究，每年召开1至2次高层次务虚会，市级领导同志每人联系1个乡（镇、街道），每年下基层时间不少于2个月，每年牵头撰写1至2篇调研报告。下大力精简会议活动，严格控制以市政府名义召开的会议或举办的活动，改进会议形式，提高会议实效，降低会议成本。大幅减少文件简报，提高质量实效。简化、改进新闻报道，减少报道数量，压减报道篇幅和时间。简化接待工作，厉行勤俭节约，禁止铺张浪费。严格执行政府工作推进“八项制度”，对每项工作都要明确责任、加强督导、严格考核、兑现奖惩。继续实行领导干部帮包重点项目制度，带动形成抓落实、求实效的良好风气。

　　（二）全面推进依法行政。自觉接受市人大及其常委会的监督，认真落实各项决议、决定并定期报告工作。积极支持人民政协履行政治协商、民主监督、参政议政职能，认真听取各民主党派、工商联、无党派人士和人民团体的意见建议。认真办理好人大代表建议和政协委员提案。深入实施依法行政“六五”规划。严格执行重大行政决策程序规定，完善政府法律顾问制度，推进依法民主科学决策。深入推进行政审批制度改革，全面清理、精减审批事项，做到行政审批应进项目100%进驻行政服务中心、可授权项目100%授权。加强对行政执法过程的监督，落实重大行政执法备案制度，促进行政行为规范化。加强电子政务建设，深化政务公开，及时公开重要政务和民生信息。抓好市长公开电话、行风热线建设和社情民意调查，完善行政复议制度，建立复议与信访衔接机制，及时解决群众的合法诉求。

　　（三）认真抓好反腐倡廉。坚持标本兼治、综合治理、惩防并举、注重预防的方针，加快推进惩治和预防腐败体系建设，切实把廉政建设的要求落实到政府工作的各个环节。坚持廉洁自律，严格遵守《中国共产党领导干部廉洁从政若干准则》，严格要求亲属和身边工作人员，严格执行住房、车辆配备等工作和生活待遇的规定。加强经济责任、“三公经费”和重点项目早期跟进审计，推进审计整改工作制度化、规范化。加强行政监察，严格执行问责办法，深入开展专项治理，严肃查处失职渎职、提供“保护伞”等违纪违法行为。加强科技防腐工作，形成覆盖市、县、乡三级的科技防腐网络，从源头上预防和惩治腐败。

　　各位代表，“空谈误国，实干兴邦”。要完成今年政府工作的各项目标任务，我们必须脚踏实地，埋头苦干，扎实工作，狠抓落实。让我们紧密团结在以习近平同志为总书记的党中央周围，高举中国特色社会主义伟大旗帜，在中共聊城市委的坚强领导下，凝聚力量、开拓创新、砥砺奋进，为全面建成小康社会、加快建设生态型强市名城、创造全市人民幸福生活而努力奋斗！
